# Treatment with midostaurin and other FLT3 targeting inhibitors is associated with an increased risk of cardiovascular adverse events in patients who underwent allogeneic hematopoietic stem cell transplantation with FLT3-mutated AML

**DOI:** 10.1007/s00277-023-05396-y

**Published:** 2023-08-08

**Authors:** Anjali Cremer, Julius C. Enssle, Saskia Pfaff, Khouloud Kouidri, Fabian Lang, Christian Brandts, Andreas Zeiher, Sebastian Cremer, Björn Steffen, Hubert Serve, Gesine Bug

**Affiliations:** 1 Department of Medicine, Hematology/Oncology, University Hospital Frankfurt, Goethe University, Frankfurt Am Main, Germany; 2 Frankfurt Cancer Institute (FCI), Frankfurt Am Main, Germany; 3 German Cancer Consortium (DKTK) and German Cancer Research Center (DKFZ), Heidelberg, Frankfurt Am Main, Germany; 4 University Cancer Center Frankfurt (UCT), University Hospital, Goethe University, Frankfurt, Germany; 5 Department of Medicine, Cardiology, University Hospital Frankfurt, Goethe University, Frankfurt Am Main, Germany

**Keywords:** Allogeneic stem cell transplantation, Acute myeloid leukemia, Targeted FLT3 inhibitor therapy, Cardiac toxicities

## Abstract

The addition of midostaurin to standard chemotherapy has improved survival in patients with *FLT3*-mutated AML. However, the impact of midostaurin and other FLT3 inhibitors (FLT3i) on cardiovascular adverse events (CAEs) has not been studied in patients who underwent allogeneic hematopoietic stem cell transplantation in a real-world setting. We reviewed 132 patients with AML who were treated with intensive induction therapy and consecutive allogeneic stem cell transplantation at our institution (42 *FLT3*-mutated AML and 90 with *FLT3* wildtype). We identified treatment with midostaurin and/or FLT3i as an independent risk factor for CAEs not resulting in higher non-relapse mortality (NRM) or impaired overall survival (OS). Hence, close monitoring for CAEs is warranted for these patients.

**Supplementary Information:**

The online version contains supplementary material available at 10.1007/s00277-023-05396-y.

## Introduction

Fms-like tyrosine kinase 3 gene (*FLT3*) is one of the most frequently mutated genes in AML detected in 30% of newly diagnosed adult patients [1]. Seventy-five percent of these patients harbor a *FLT3* internal tandem duplication (*ITD*), which results in the duplication of 3 or more amino acids located in the juxtamembrane region; fewer patients have a *FLT3* point mutation in the tyrosine kinase domain (*TKD*) [2]. *FLT3-ITD* mutations especially with high mutant to wildtype (mutant/wild type) allelic ratio [3] are associated with an unfavorable prognosis [4] due to a particularly high risk of AML relapse and leukemic death despite treatment with allogeneic hematopoietic stem cell transplantation (HSCT) [3, 5]. The prognostic impact of *FLT3-TKD* mutations is less clear [6, 7]. Superior outcome has been achieved in recent years by integrating FLT3 inhibitors into the treatment algorithm of *FLT3*-mutated AML. Addition of the multikinase inhibitor midostaurin to standard chemotherapy in patients with a *FLT3*-mutated AML led to a longer overall and event-free survival [2]. Eligible patients with *FLT3-ITD*^*high*^* or FLT3-ITD*^*low*^* NPM1* wildtype AML benefitted from an allogeneic HSCT [8], followed by post-transplant maintenance with sorafenib [9, 10]. The more specific FLT3 inhibitors gilteritinib [11] and quizartinib [12] were used for salvage treatment.

Cardiovascular complications are common among patients with acute leukemia after treatment with chemotherapeutic agents and have also been reported for selective tyrosine as well as multitarget kinase inhibitors. One of the most common side effects observed in the safety evaluation of FLT3 inhibitors was prolongation of the QTcF (corrected QT interval using Friderica’s formula) interval [11, 12]. However, other cardiac complications caused by these drugs have not been studied in detail in patients in a real-world setting, including patients who underwent allogeneic HSCT but have been identified as potential reasons for discontinuation of treatment [13].

The aim of this retrospective single-center study was to evaluate the frequency of and identify risk factors for cardiac adverse events (CAEs) in consecutively transplanted AML patients treated with midostaurin and/or other FLT3 targeting agents.

## Methods

This retrospective analysis included all patients with newly diagnosed secondary or de novo AML who received an intensive induction chemotherapy and underwent allogeneic HSCT at our center between January 2017 and April 2021. The primary endpoint was the occurrence of a cardiac adverse event.

The indication for allogeneic HSCT was performed based on general practice considerations including the ELN 2017 risk classification or poor response after the first cycle of induction assessed by bone marrow aspiration on day 15. The majority of patients received a fludarabine and melphalan-based conditioning regimen before allogeneic HSCT.

Midostaurin was administered in a standard dose of 50 mg every 12 h from days 8–21 as recommended by the pharmaceutical provider and were discontinued 48 h before conditioning for allogeneic stem cell transplantation. Sorafenib was administered at a dose of up to 400 mg twice daily as recommended in the manufacturer’s information. Quizartinib was administered at a 60 mg dose (with a 30 mg lead in) as described in the Quantum-R trial [14]. Gilteritinib was administered at 120 mg once daily as described in the manufacturer’s information.

Clinical data were retrieved from patient records including preexisting cardiac comorbidities and CAEs, i.e., coronary artery disease, heart failure, valvular heart disease, arrhythmias, and reduced left ventricular ejection fraction (LVEF ≤ 50%) [15].

For statistical analysis, R version 4.1.2 (the R Foundation for Statistical Computing, R Core Team, Vienna, Austria) was used. Data are presented either as categorical variables and were analyzed with Chi-square or Fisher’s *t*-test or continuous data and analyzed by Wilcoxon rank sum or Kruskal–Wallis test, where appropriate. Uni- and multivariate analysis was performed by logistic regression. Statistical significance was determined at *P* < 0.05. Overall survival (OS) was calculated from diagnosis. Cumulative incidence (CI) was used to estimate the incidence of cardiac events with death as competing risk. The median follow-up of surviving patients was 36.5 (3–77) months. Patient outcome was reported at 36 months after treatment initiation. All procedures followed were in accordance with the ethical standards of the responsible committee on human experimentation (institutional and national) and with the Helsinki Declaration of 1975, as revised in 2008. The study was approved by the institutional ethic’s review board, and all patients provided informed consent for use of their medical record data for research (SNH-7–2021) (Table 1).

**Table 1:** Patient characteristics

	Overall	No Mido/FLT3i	Mido/FLT3i	*p*-value
*n*	132	98	34	
Female, *n* (%)	50 (37.9)	32 (32.7)	18 (52.9)	0.058
Age, median [IQR]	56.00 [46.00, 64.25]	56.50 [46.50, 64.75]	52.50 [46.25, 63.00]	0.617
FLT3 mutation status, *n* (%)				< 0.001
ITD	35 (26.5)	6 (6.1)	29 (85.3)	
TKD	4 (3.0)	1 (1.0)	3 (8.8)	
ITD + TKD	3 (2.3)	1 (1.0)	2 (5.9)	
WT	90 (68.2)	90 (91.8)	0 (0.0)	
VAF ratio, *n* (%)				0.804
High	20 (47.6)	3 (37.5)	17 (50.0)	
Low	17 (40.5)	4 (50.0)	13 (38.2)	
NA	5 (11.9)	1 (12.5)	4 (11.8)	
NPM1 status, *n* (%)				< 0.001
mut	36 (27.3)	16 (16.3)	20 (58.8)	
WT	93 (70.5)	79 (80.6)	14 (41.2)	
NA	3 (2.3)	3 (3.1)	0 (0.0)	
ELN classification, *n* (%)				0.109
Favorable risk	26 (19.7)	16 (16.3)	10 (29.4)	
Intermediate risk	67 (50.8)	49 (50.0)	18 (52.9)	
Adverse risk	39 (29.5)	33 (33.7)	6 (17.6)	
Type of alloHSCT, *n* (%)				0.711
Haplo	17 (12.9)	14 (14.3)	3 (8.8)	
HLA-ident	108 (81.8)	79 (80.6)	29 (85.3)	
Partially HLA-ident	7 (5.3)	5 (5.1)	2 (5.9)	
Induction cycles, *n* (%)				0.047
1	54 (40.9)	42 (42.9)	12 (35.3)	
2	76 (57.6)	56 (57.1)	20 (58.8)	
3	2 (1.5)	0 (0.0)	2 (5.9)	
Obese (BMI ≥ 30), *n* (%)	18 (14.1)	12 (12.6)	6 (18.2)	0.617
Sorror Score, *n* (*n*%)				0.349
0	41 (31.1)	31 (31.6)	10 (29.4)	
1	20 (15.2)	19 (19.4)	1 (2.9)	
2	16 (12.1)	11 (11.2)	5 (14.7)	
3	29 (22.0)	18 (18.4)	11 (32.4)	
4	11 (8.3)	9 (9.2)	2 (5.9)	
5	8 (6.1)	5 (5.1)	3 (8.8)	
6	4 (3.0)	3 (3.1)	1 (2.9)	
7	2 (1.5)	1 (1.0)	1 (2.9)	
8	1 (0.8)	1 (1.0)	0 (0.0)	
Cardiac event, *n* (%)	39 (29.5)	19 (19.4)	20 (58.8)	< 0.001
≥ Grade 3 cardiac event, *n* (%)	20 (15.2)	12 (12.2)	8 (23.5)	0.192
1st cardiac event pre alloHSCT, *n* (%)	15 (11.4)	7 (7.1)	8 (23.5)	0.023
Cardiac premorbidity, *n* (%)	22 (16.7)	18 (18.4)	4 (11.8)	0.533
Baseline hypertension, *n* (%)	38 (28.8)	30 (30.6)	8 (23.5)	0.571

*alloHSCT*, allogenic hematopoietic stem cell transplantation; *BMI*, body mass index; *ELN*, European Leukemia Network; *FLT3i*, FLT3 inhibitor; *IQR*, interquartile range; *ITD*, internal tandem duplication; *mut*, mutated; *TKD*, tyrosine kinase domain

## Results and discussion

We screened 318 patients who underwent allogeneic HSCT between 1/2017 and 4/2021 at our institution. Next, we identified 162 patients with an AML (de novo or secondary) and included 132 patients in our study who were treated with intensive induction chemotherapy and consecutive allogeneic HSCT (Supplemental Fig. 1): 90 patients (68.2%) with *FLT3* wildtype; 42 patients with mutated *FLT3*, i.e., a *FLT3-ITD* (*n* = 35, 26.5%); a *FLT3-TKD* mutation (*n* = 4, 3%); or both mutations (*n* = 3, 2.3%). The median age was 56 years (IQR 46–65). Of the 42 patients harboring a *FLT3* mutation, 34 patients (82%) received a FLT3 inhibitor at some time during their treatment: 28 patients (82.35%) received midostaurin, 24 patients (70.6%) received another FLT3i (sorafenib *n* = 18, 75.0%, or sequential treatment of sorafenib followed by another FLT3i, e.g., quizartinib or gilteritinib; for details, see Table 2 parts a and b), and in 18 patients (52.94%), midostaurin was followed by treatment with another FLT3 targeting tyrosine kinase inhibitor. Midostaurin (*n* = 28) was administered in parallel to intensive induction chemotherapy before allogeneic HSCT, and sorafenib was used as maintenance therapy after allogeneic HSCT in 24 patients, followed by gilteritinib and/or quizartinib after MRD relapse. One patient received quizartinib for AML relapse/refractory AML prior to HSCT and sorafenib maintenance after HSCT (Table 2 parts a and b). Eight patients who harbored a mutation in the *FLT3* gene did not receive treatment with midostaurin or another FLT3 inhibitor mainly due to reimbursement issues.

**Table 2:** Distribution of inhibitors and non-Mido-FLT3i

Drug	*N* patients	% patients
a Inhibitors		
Midostaurin	28	82.35
FLT3i	24	70.59
Midostaurin and FLT3i	18	52.94
b Non-Mido-FLT3i		
Sorafenib	18	75.00
Sorafenib, gilteritinib, quizartinib	2	8.33
Sorafenib, gilteritinib	2	8.33
Quizartinib, sorafenib	1	4.17
Sorafenib, quizartinib	1	4.17

For this analysis, we compared two patient groups: one including all intensively induced AML patients, who underwent allogeneic HSCT and had received midostaurin and/or another FLT3 inhibitor (“Mido/FLT3i,” *n* = 34) during their treatment course and a second group without midostaurin or FLT3i therapy (“no Mido/FLT3i,” *n* = 98).

Patient cohorts were well balanced regarding age and obesity, whereas a slightly higher number of patients in the “no Mido/FLT3i” group had preexisting cardiac comorbidities (*n* = 18/98, 18.4% vs. *n* = 4/34, 11.8%) and hypertension at baseline (*n* = 30/98, 30.6% vs. *n* = 8/34, 23.5%), as well as a higher number of patients belonging to the intermediate (*n* = 49/98, 50% vs. 18/34, 52.9%) or adverse risk group (33/98, 33.7% vs. 6/34, 17.6%) according to ELN 2017 genetic classification [3] (Table 1).

Prediction of transplant related mortality (TRM), including cardiac comorbidities, was assessed by application of the hematopoietic cell transplantation (HCT)-specific comorbidity index (Sorror score) [15] (Table 1). Both groups were balanced regarding the Sorror score and did not show a skewing of higher Sorror scores in the “Mido/TKI” group (*p* = 0.531). Patient and treatment characteristics are described in detail in Table 1. Overall, 39/132 patients (29.5%) developed a cardiac adverse event (CAE) after starting their antileukemic treatment. Importantly, the majority (*n* = 20/34, 58.8%, *p* < 0.001) belonged to the “Mido/FLT3i” group who received midostaurin or another FLT3 inhibitor during their treatment, while 19/98 patients (19.4%) belonged to the “no Mido/FLT3i” group (Table 1).

Most of the documented CAEs in the “Mido/FLT3i” group were mild according to common terminology criteria of adverse events (CTCAE) (grade 1–2, Supplemental Table S1).

The most frequent observed CAE in the “Mido/FLT3i” group was heart failure (Supplemental Table S1), while in the “no Mido/FLT3i” group, it was heart failure and left ventricular systolic dysfunction (Supplemental Table S1). In the MidoFLT3i group, eight patients (23.5%) developed a ≥ grade 3 CAE, while this was observed in 12 patients (12.2%) in the “no MidoFLT3i” group (*p* = 0.192). There were five patients (14.7%) who developed more than one grade 3–5 event in the “Mido/FLT3i” group, and one of these patients died of cardiac toxicity after midostaurin treatment, while only two patients in the “no MidoFLT3i” group exhibited ≥ 1 grade 3–5 CAE (2%,* p* = 0.0166) (Supplemental Table S1 and S2). This suggests a tendency towards more frequent severe cardiac toxicities in the patient cohort who received treatment with a FLT3i (Supplemental Table 1). However, larger patient cohorts will be needed to evaluate this in more detail. Regarding the onset time of observed first CAEs, eight patients (23.5%) in the “MidoFLT3i” group developed a CAE before HSCT (who all received Midostaurin) compared to seven patients (7.1%) in the “no MidoFLT3i” group (*p* < 0.023, Table 1). This highlights the association of midostaurin or FLT3i therapy with increased frequencies of CAEs already during induction treatment.

In four patients, FLT3i maintenance therapy was discontinued due to adverse effects (skin rash *n* = 2, hypertension *n* = 1, liver failure *n* = 1). In one patient, sorafenib in combination with induction chemotherapy as a first-line treatment was continued with a lower dose after registration of abnormally inverted T waves in an ECG, which resolved after dose reduction.

**Fig. 1:**
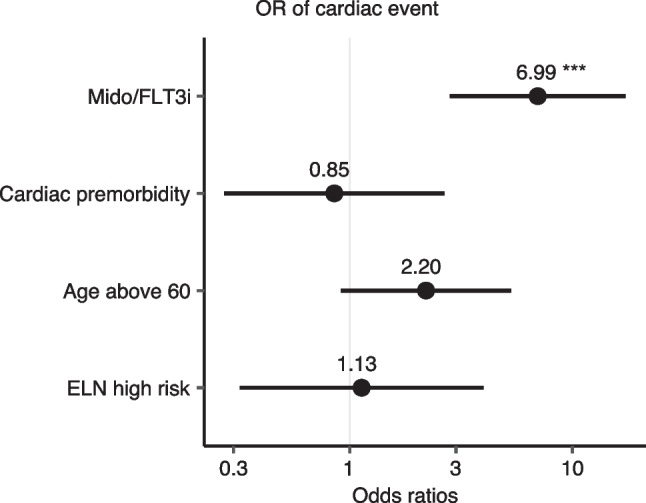
Forest plot of odd’s ratios (OR) of cardiac events according to subgroups. Odds ratios are reported numerically, *p*-values are highlighted with *** < 0.001

By univariate analysis, only treatment with midostaurin and/or FLT3 inhibitor was associated with a statistically significant risk for CAEs (OR 5.94 [97.5% CI 2.58–14.16], *p* < 0.001) (Table 3). Other known risk factors (i.e., age ≥ 60 years, ELN adverse or intermediate risk, obesity, preexisting cardiac comorbidity, female sex, two induction cycles, or cumulative daunorubicin equivalence dose) were not associated with a significantly higher number of CAEs (Table 3). When we performed multivariate regression analysis, we confirmed treatment with midostaurin and/or FLT3i as an independent risk factor for new CAEs (OR 6.99 [97.5% CI 2.89–18.03], *p* < 0.001; Fig. 1, Table 4).

**Table 3:** Univariate analysis

Variable	OR	2.5% CI	97.5% CI	*p*-value
Midostaurin and/or FLT3i	5.94	2.58	14.16	< 0.001
Age ≥ 60	1.56	0.73	3.34	0.25
ELN adverse risk	0.78	0.26	2.38	0.65
ELN intermediate risk	1.03	0.39	2.84	0.96
2 induction cycles	1.12	0.53	2.47	0.76
Obesity (BMI ≥ 30)	0.26	0.04	0.97	0.08
Cardiac premorbidity	0.88	0.29	2.34	0.80
Female sex	1.40	0.65	3.01	0.38
Cumulative daunorubicin equivalence dose	1.00	1.00	1.01	0.42

*BMI*, body mass index; *ELN*, European Leukemia Network; *FLT3i*, FLT3 inhibitor; *OR*, odds ratio

**Table 4:** Multivariate Analysis

Variable	OR	2.5% CI	97.5% CI	*p*-value
Midostaurin and/or FLT3i	6.99	2.89	18.03	< 0.001
Cardiac premorbidity	0.85	0.25	2.57	0.78
Age ≥ 60	2.20	0.92	5.44	0.08
ELN 2017 adverse risk	1.13	0.32	4.13	0.85
ELN 2017 intermediate risk	1.24	0.42	3.91	0.70

*BMI*, body mass index; *ELN*, European Leukemia Network; *FLT3i*, FLT3 inhibitor; *OR*, odds ratio

Seventeen of all 132 (12.9%) patients received an allogeneic HSCT from a haploidentical donor using post-transplant cyclophosphamide (PTCy) as graft-versus-host-disease (GVHD) prophylaxis (Table 1). The administration of PTCy in the haploidentical transplantation setting is known to cause cardiotoxicity [16]. However, the percentage of patients receiving PTCy was similar in both groups (“no Mido/FLT3i,” 14.3%; “Mido/FLT3i” 8.8%), suggesting no significant contribution to the increased observation of cardiac events in the “Mido/FLT3i” group.

The cumulative incidence estimate (CIE) of CAEs was significantly higher in the “Mido/FLT3i” group compared to the “no Mido/FLT3i” group (57.6% vs. 17.8%, *p* < 0.001, supplemental fig. S2, black lines) at 36 months. Of note, cardiac toxicity was not associated with a higher CIE of non-relapse mortality (NRM) in the “MidoFLT3i “ group compared to the “no MidoFLT3i “ group (6.4% vs. 11.1% *p* = 0.51, supplemental fig. S3) or a difference in OS with an estimate of 59.2% surviving patients in the “MidoFLT3i” group vs. 70.8% in the “no MidoFLT3i” group (*p* = 0.94, supplemental fig. S4) at 36 months.

In line with our observation, a recent abstract of a World Health Organization (WHO) pharmacovigilance database meta-analysis [17] suggested an increase of CAEs (QT prolongation, heart failure, atrial fibrillation, and pericardial disease) in patients who received midostaurin, corroborating the observations from our patient cohort [17]. According to our data, monitoring for CAEs should be performed at least for the duration of FLT3i treatment by ECGs and transthoracic echocardiograms (TTE). In case of CAEs, FLT3i need to be dose-reduced under close cardiac monitoring of the patient or discontinued. The findings of our study are limited by the small patient cohort. Not all CAEs of grade 1–2 can be captured by clinical monitoring only. We have previously established standard operating procedures (SOPs) including cardiovascular clinical examination, ECG, and TTE before HSCT and at day 100, 1 year, 2 years for all our patients after HSCT, or more often in case of an abnormal finding. Therefore, both groups have been monitored for CAEs similarly, except that we have more records of ECGs in patients from the “Mido/FLT3i” group.

In summary, we observed a higher incidence of mostly mild grade 1–2 CAEs in patients with newly diagnosed AML, receiving intensive induction therapy and allogeneic HSCT, and were treated with midostaurin or another FLT3 targeting inhibitor in comparison to patients who did not receive midostaurin or another FLT3i. However, the observed CAEs did not have an impact on NRM or OS. To the best of our knowledge, our study represents the first real-world data demonstrating an association of CAEs in patients who have been treated with midostaurin or another FLT3 targeting inhibitor. The higher risk of rarely life-threatening CAE should not preclude administration of midostaurin and/or FLT3i, even in patients with preexisting cardiac comorbidities, but close monitoring is warranted.

## Supplementary Information

Below is the link to the electronic supplementary material.

Supplementary file1 (PDF 92 KB)

Supplementary file2 (PDF 87 KB)

Supplementary file3 (PDF 13 KB)

Supplementary file4 (PDF 13 KB)

Supplementary file5 (DOCX 23 KB)

Supplementary file6 (XLSX 15 KB)

## Data Availability

Data will be made available upon reasonable request to the corresponding author.
