# Supplementary figures and images for: Treatment with midostaurin and other FLT3 targeting inhibitors is associated with an increased risk of cardiovascular adverse events in patients who underwent allogeneic hematopoietic stem cell transplantation with FLT3-mutated AML
Source: Ann Hematol. 2023 Aug 8;102(10):2903–8. doi: 10.1007/s00277-023-05396-y (PMC10492676; doi:10.1007/s00277-023-05396-y)

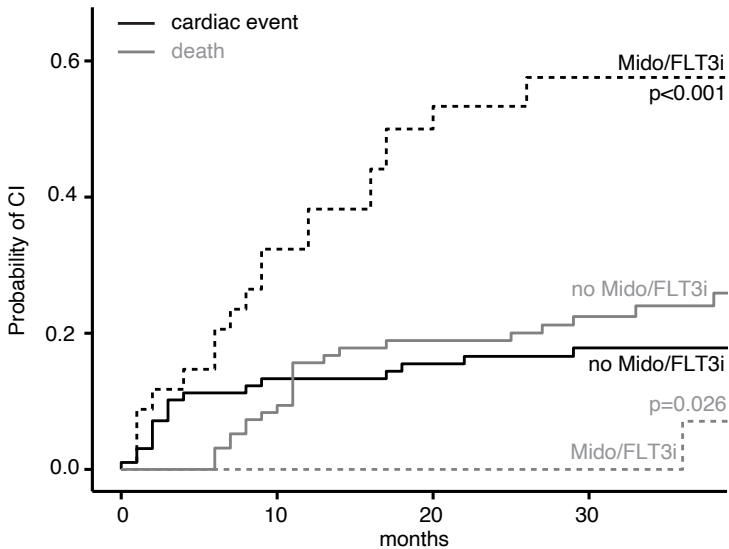

Supplement: Supplementary file 1 — Supplementary file1 (PDF 92 KB) [file 277_2023_5396_MOESM1_ESM.pdf]

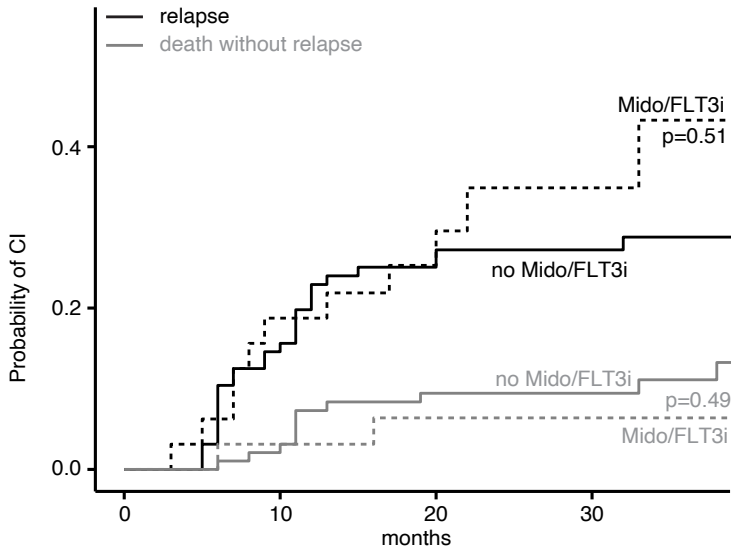

Supplement: Supplementary file 2 — Supplementary file2 (PDF 87 KB) [file 277_2023_5396_MOESM2_ESM.pdf]

Probability of overall survival

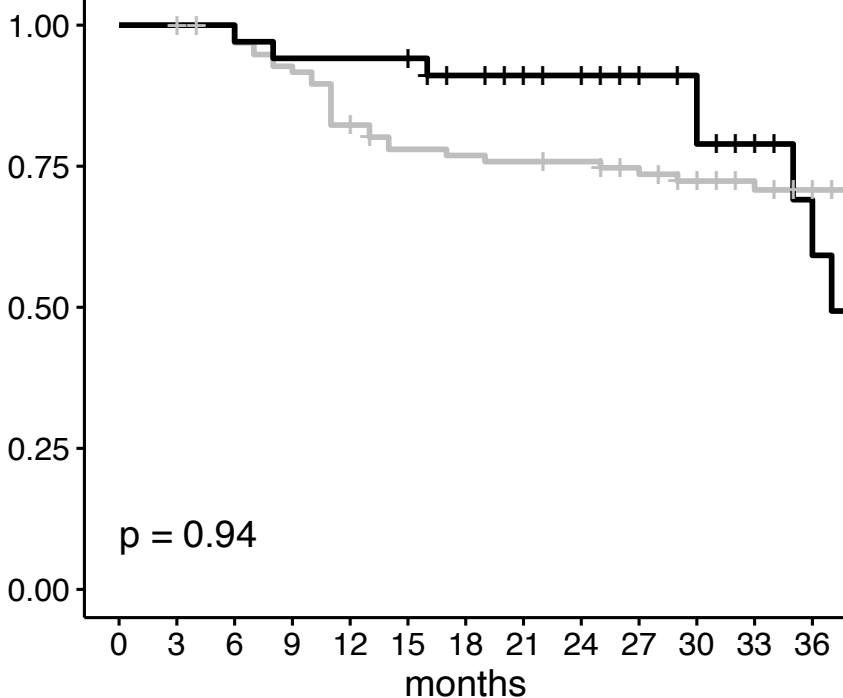

No. at risk

no Mido/FLT3i

98 98 96 89 79 72 71 70 69 65 54 46 42

Mido/FLT3i

34 34 34 32 32 32 27 23 21 17 15 11 7

0 3 6 9 12 15 18 21 24 27 30 33 36

months

Supplement: Supplementary file 3 — Supplementary file3 (PDF 13 KB) [file 277_2023_5396_MOESM3_ESM.pdf]

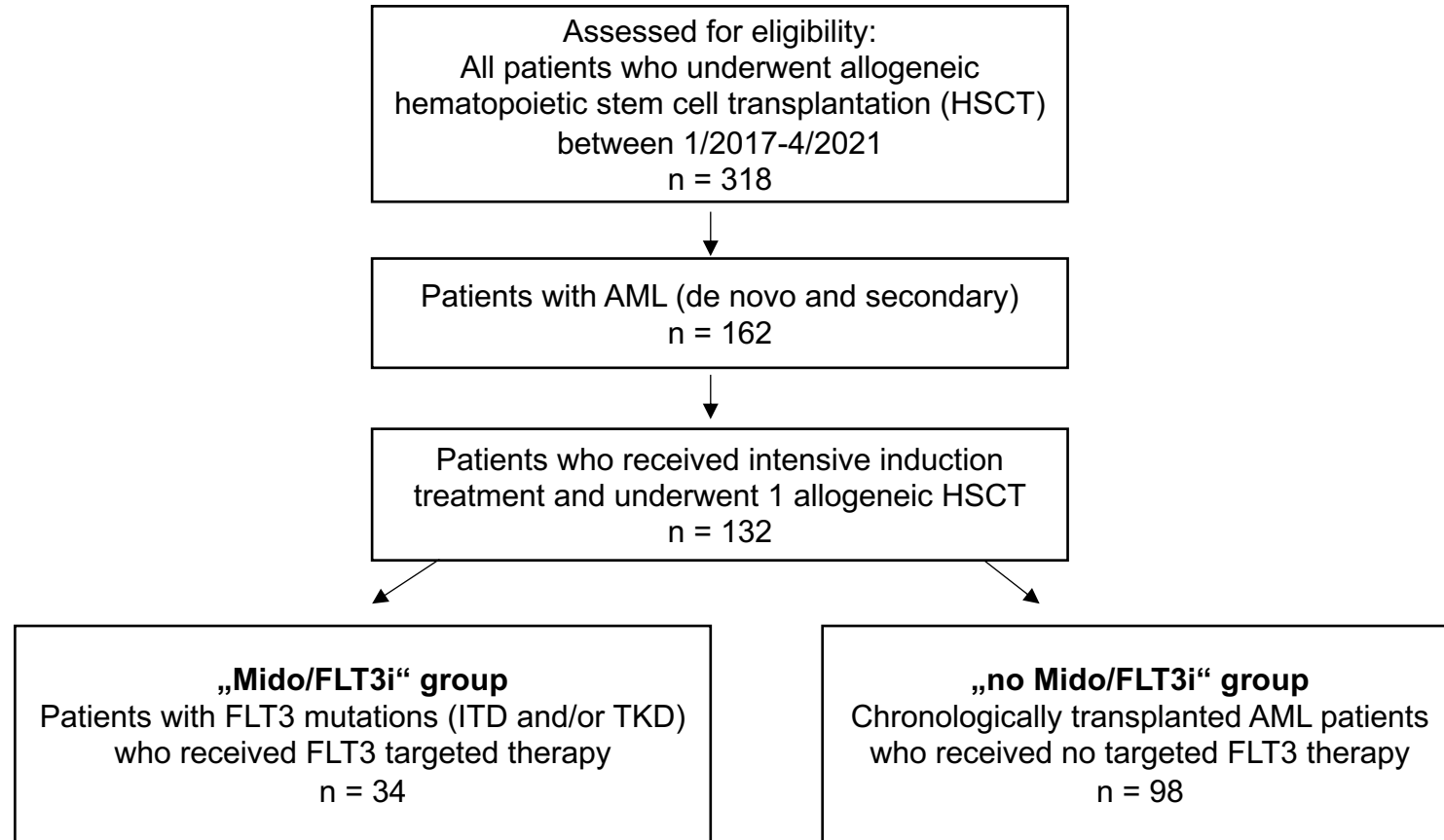

Supplement: Supplementary file 4 — Supplementary file4 (PDF 13 KB) [file 277_2023_5396_MOESM4_ESM.pdf]
