# Supplementary material for: Treatment with midostaurin and other FLT3 targeting inhibitors is associated with an increased risk of cardiovascular adverse events in patients who underwent allogeneic hematopoietic stem cell transplantation with FLT3-mutated AML
Source: Ann Hematol. 2023 Aug 8;102(10):2903–8. doi: 10.1007/s00277-023-05396-y (PMC10492676; doi:10.1007/s00277-023-05396-y)
Supplement: Supplementary file 5 — Supplementary file5 (DOCX 23 KB) [file 277_2023_5396_MOESM5_ESM.docx]

Supplemental Methods

Genetic analysis

Screening for FLT3 mutations was performed at the Münchner Leukämie Labor (MLL) reference laboratory (München, Germany), using the standardized assay^1^. An *FLT3-ITD* allelic ratio ≥ 0.05, as determined by DNA fragment analysis, was considered positive. *FLT3 TKD* mutations were also analyzed as previously described^2^.

Statistical analysis

The R version 4.1.2 was used for statistical analysis^3^. Competing risk analysis was performed with the cmprsk package^4^.

References for supplemental methods

1. Schnittger S, Schoch C, Dugas M, et al. Analysis of FLT3 length mutations in 1003 patients with acute myeloid leukemia: correlation to cytogenetics, FAB subtype, and prognosis in the AMLCG study and usefulness as a marker for the detection of minimal residual disease. *Blood*. 2002;100(1):59-66.

2. Bacher U, Haferlach C, Kern W, Haferlach T, Schnittger S. Prognostic relevance of FLT3-TKD mutations in AML: the combination matters--an analysis of 3082 patients. *Blood*. 2008;111(5):2527-2537.

3. Team RC. R: A language and environment for statistical computing. R Foundation for Statistical Computing, Vienna, Austria. 2021.

4. Scrucca L, Santucci A, Aversa F. Competing risk analysis using R: an easy guide for clinicians. *Bone Marrow Transplant*. 2007;40(4):381-387.

Supplemental Figure Legends

Supplemental Figure S1: Consort diagram describing how we identified patients for our study and allocated them to the “Mido/FLT3i” and “No Mido/FLT3i” group.

Supplemental Figure S2: Cumulative incidence (CI) estimate for cardiac events (p<0.001 for MidoFLT3i vs. no Mido/FLT3i) with death as competing risk (p=0.026 for MidoFLT3i vs. no Mido/FLT3i). Black denotes the cardiac event and grey the competing risk (death). Dotted lines indicate the CI estimate for the MidoFLT3i group while straight lines highlight the no Mido/FLT3i group.

Supplemental Figure S3:

Cumulative incidence (CI) estimate for relapse (p=0.51 for MidoFLT3i vs. no Mido/FLT3i) with death without relapse as competing risk (p=0.49 for MidoFLT3i vs. no Mido/FLT3i). Black denotes relapse and grey the competing risk (death without relapse). Dotted lines indicate the CI estimate for the MidoFLT3i group while straight lines highlight the no Mido/FLT3i group.

Supplemental Figure S4: Kaplan Meier analysis depicting the overall survival (OS) probability stratified for Mido/FLT3i versus no Mido/FLT3i. P-value was obtained by log-rank test.
